# Thermal Insulation Efficiency and Mechanisms of Flexible Sandwich Structure

**DOI:** 10.3390/ma18071426

**Published:** 2025-03-24

**Authors:** Yuanzhe Xie, Juan Chen, Shuo Li, Mengxiao Guo, Niansu Jing

**Affiliations:** School of Materials Science and Engineering, University of Jinan, Jinan 250022, China; xyzkrystal0919@163.com (Y.X.); hechengli8845@gmail.com (S.L.); guomengxiao2022@163.com (M.G.); jns13969123166@163.com (N.J.)

**Keywords:** mica roll, ceramic fiber felt, composite sandwich structure, cold-surface temperature rise, numerical simulation

## Abstract

Thermal insulation layers between batteries are an effective way to reduce the propagation of thermal runaway in lithium-ion batteries. A flexible composite sandwich structure material has been designed for thermal insulation, featuring mica rolls (MRs) as the protective layers and a ceramic fiber felt (CFF) as the core layer. Experimental and numerical simulations demonstrate that at a hot-surface temperature of 900 °C, the cold-surface temperature of the sandwich structure with a 0.3 mm MR and 3.0 mm CFF layer is only 175 °C, which is significantly lower than the 350 °C observed for a standalone 3.0 mm CFF layer under the same conditions. The MR layer effectively shields against flames and impedes heat transfer, while the porous structure of the CFF, enhanced by microcracks and holes, increases heat transfer paths and scatters radiated heat. This synergistic interaction between the MR and CFF layers results in a superior thermal insulation performance. The findings highlight the potential of this sandwich structure in improving the safety and thermal management of lithium-ion batteries and other energy storage systems.

## 1. Introduction

The advent of lithium-ion batteries has revolutionized the field of energy storage, underpinning the growth of electric vehicles and a range of portable electronics. However, the high energy density of these batteries brings forth significant thermal management challenges. The risk of thermal runaway, a phenomenon where internal heat generation leads to uncontrollable temperature increases, poses a significant safety concern [1]. This issue is exacerbated in large-format batteries, whose energy storage capacity and potential for thermal cascade make effective thermal insulation imperative [2].

Strategies to mitigate thermal runaway have focused on enhancing the thermal stability of battery components and controlling the thermal environment within the battery pack. Of these, the incorporation of a thermal insulation layer [3,4,5,6] between battery modules has emerged as a critical approach to contain thermal runaway and prevent its propagation to adjacent cells [6].

In addressing these challenges, the scientific community has made significant strides in developing materials and systems designed to manage and dissipate heat effectively. Yuan et al. [7] investigated the inhibition effect of different interstitial materials on thermal runaway propagation in cylindrical LIB modules, highlighting the significance of thermal path control in the safe design of batteries. Similarly, Sun et al. [8] explored the effects of thermal insulation layer materials on thermal runaway in energy storage LIB packs, demonstrating the potential of using phase-change materials to achieve zero spreading of thermal runaway within the module. The use of aerogel-based insulation materials has shown promise in enhancing the thermal stability of LIBs. Liu et al. [9] conducted experiments to mitigate thermal runaway propagation in overcharged lithium-ion battery modules using various thermal insulation layers, emphasizing the efficacy of fiber-based and aerogel materials in enhancing thermal management and safety. Yu et al. [2] developed a sandwich structured ultra-strong-heat-shielding aerogel/copper composite insulation board for LIB modules, showcasing its effectiveness in reducing heat transfer through convection, conduction, and radiation. This approach aligns with the findings of Kovács et al. [10], who investigated the thermal stability of different aerogel insulation materials at elevated temperatures, emphasizing the need for materials that maintain their insulating capabilities under high-temperature conditions. The mechanical support framework provided by hollow glass microspheres (HGMs) and the thermal insulation performance conferred by aerogel particles (APs) have been combined in a novel insulation material designed to mitigate TRP in LIBs, which was presented by Yin et al. [11]. This material not only exhibits exceptional compressive strength and flame retardancy but also demonstrates a high thermal insulation performance, making it a promising candidate for enhancing the safety of LIB modules. Wang et al. [12] discussed an improved sandwich-structured composite with low thermal conductivity used in high-temperature applications, which has great potential for use in thermal protection systems. This composite, composed of a compound core material and C/SiC laminates, demonstrated significantly lower thermal conductivity than unfilled SiC foam at high temperatures. The compound core material, made of reticulated SiC foam and SiO_2_ powders, effectively controlled thermal radiation at high temperatures, leading to excellent thermal insulation performance. Du et al. [13] have opened up new avenues for battery protection. Inspired by the hierarchical structure of wood, they designed and fabricated a novel protective shielding material which possesses superior mechanical robustness and flame-retardant properties, effectively mitigating thermal runaway while also offering sufficient strength to protect batteries from mechanical abuse.

Building on the material advancements for thermal management in lithium-ion batteries (LIBs), researchers have also employed numerical simulations to explore and optimize thermal control strategies [2,14,15,16,17,18]. These simulations provide a cost-effective and time-efficient means of predicting the thermal behavior of LIBs under various conditions and configurations, allowing for the testing of different thermal insulation designs before physical prototyping. In this context, Yan et al. [16] conducted a numerical study of the thermal performance of a composite board in battery thermal management systems (BTMSs). They designed a composite board with a sandwich structure for use in BTMSs, aiming to balance its heat dissipation and insulation capabilities. Through detailed comparisons of four different operating modes under normal and thermal abuse conditions, they demonstrated that the composite board could effectively improve heat dissipation and temperature uniformity while enhancing the heat insulation capability of the battery pack. Building on this, the numerical simulations in Yu’s [2] work supported experimental findings, showing a significant reduction in temperature rise and heat transfer rates with the protection of the STI board.

In the pursuit of enhanced thermal insulation between battery cells, researchers have dedicated significant efforts to studying the heat insulation mechanisms of various materials. Feng et al. [19] highlighted radiative heat attenuation mechanisms by calculating thermal radiation optical parameters including scattering, absorption, and attenuation factors. They discovered that the incorporation of SiC as an opacifier significantly enhances the thermal insulation performance of systems at elevated temperatures, particularly where radiative heat transfer becomes the predominant factor within the entire heat transfer system. Guo et al. [20] developed ultra-flexible TiO_2_/SiO_2_ nanofiber membranes with a layered structure for thermal insulation. The layered structure, based on the principle of multi-level reflection, enhances the near-infrared reflection effect, demonstrating an excellent comprehensive thermal insulation effect by reducing hot-surface temperatures significantly. Wan et al. [21] have provided further depth to our understanding of thermal insulation mechanisms. Their two-flux radiative heat transfer model built for fibrous assemblies with reflective interlayers has optimized the construction of fibrous insulation, maximizing overall thermal insulation by balancing conductive and radiative heat transfers.

Although layered structural materials exhibit excellent heat insulation properties, their practical applications are still limited. Many layered materials have poor thermal stability at high temperatures and are prone to structural change or decomposition [22,23]. In addition, some layered materials are not mechanically strong enough to maintain stable performance under harsh conditions. The material mica has excellent mechanical properties and a heat shielding ability [20]; its unique layered structure increases the heat conduction path and changes the direction of heat flow [24,25], while its interlayer thermal resistance further blocks heat transfer, making its range of applications more and more extensive.

In this paper, a flexible composite sandwich structure (MR//CFF//MR) is designed for thermal insulation, with mica rolls (MRs) as the protective layer and a ceramic fiber felt (CFF) as the core layer. The cold-surface temperature of the material was simulated and predicted by Abaqus numerical simulation. Through a series of experiments and numerical simulations, the thermal insulation efficiency and mechanisms of the composite have been thoroughly investigated. This sandwich structure facilitates a synergistic heat transfer effect for superior insulation. The flexible mica protective layer shields against flames and blocks heat transfer, enhancing the insulation provided by the CFF’s porous structure through its microcracks and holes, which increase heat transfer paths and scatter radiated heat.

## 2. Experimental Procedure

### 2.1. Materials and Characterization

The mica roll (MR) was supplied by Goode Electrical Systems Co., Ltd. (Suzhou, China). The ceramic fiber felt (CFF) and the fiberglass aerogel (FA) were purchased from Morgan Thermal Ceramics (Shanghai, China) Co., Ltd. The density and thermal properties of the MR, FA, and CFF are provided in Table 1. The temperature on the cold side was tested by applying heat to the other side of the samples using a butane flame, with the heat source maintained at 900 °C, and the test lasted for 5 min. The thermogravimetric analysis (TGA) of experimental materials was performed using the instrument TG 209 F3 Tarsus (NETZSCH, Selb, Germany). The heating rate was 10 °C/min and the maximum temperature was 900 °C. The microstructure of the samples were observed by a United States FEI QUANTA FEG 250 (FEI, Hillsboro, OR, USA) thermal field emission scanning electron microscope (SEM). The test voltage was 5.00 KV. The surface functional groups of the materials were measured by an FT-IR spectrum analyzer Nicolet380 from the United States Thermal Power Company (Waukesha, WI, USA). The scanning was performed 32 times using the KBr method.

### 2.2. The Designing of Sandwich Structure Composite

**Figure 1:**
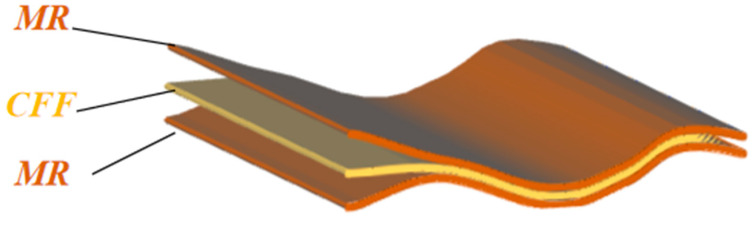
Mica//ceramic fiber felt//mica flexible sandwich structure diagram.

With MR as the protective layer and CFF as the core layer, the flexible sandwich structure (FSS) was assembled as shown in Figure 1. The thickness of each layer and the total thickness of the composite structure are shown in Table 2.

## 3. Numerical Simulation of Temperature Variations on the Cold Side Surface

The simulation of the cold side’s surface temperature variation was conducted using ABAQUS (https://www.3ds.com/products/simulia/abaqus, accessed on 10 March 2025), which took into account the properties of the materials, such as their density and specific heat capacity, and the temperature field. In this context, heat losses during the testing process and the impact of the material’s internal structure on temperature were collectively accounted for within its equivalent thermal conductivity. The physical property parameters required for the numerical simulation are presented in Table 1.

**Figure 2:**
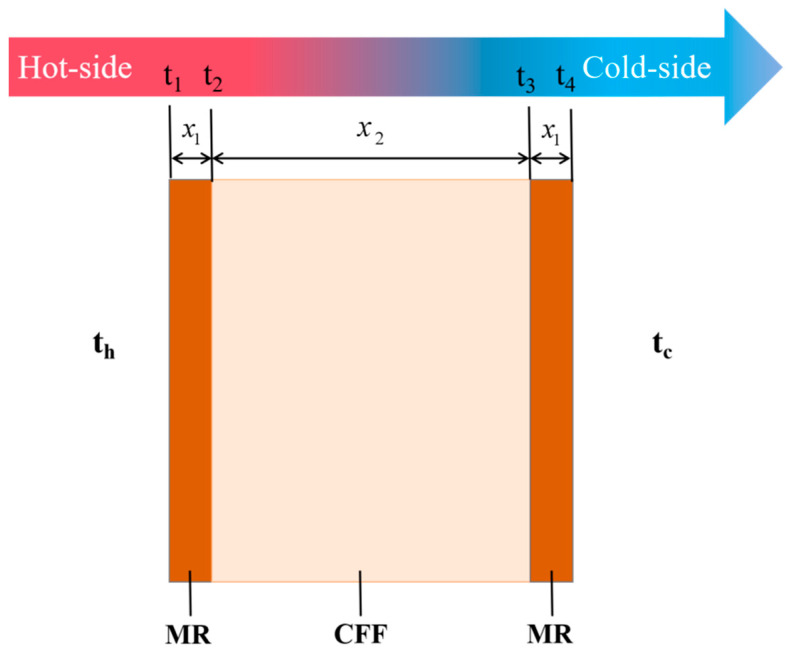
Diagram of FSS geometry.

Abaqus uses the finite element method (FEM) to numerically solve the heat conduction problem. The finite element method solves the temperature field distribution of the system through iterative calculations by discretizing the continuum into a series of interconnected elements, such as meshes, and applying heat conduction equations and boundary conditions to each element [18,26,27,28,29,30,31,32]. The geometry involved is shown in Figure 2.

Depending on the application, in the heat transfer model the contact thermal resistance of the surface and the interlaminar surface can be ignored. Given that the initial temperature of the whole system is 30 °C, the convective heat transfer temperature of the hot surface is set at 900 °C and 650 °C, respectively, while the emissivity of the mica material is 0.75 and the emissivity of the FA and CFF is 0.5. Heat transfer in a composite sandwich structure is often described as the sum of contributions from gas conduction, solid conduction, radiation, and convection, so the heat flux through the composite structure can be written as shown in Equations (1)–(3):

(1) $$q=k_{1}\frac{\left(t_{2}-t_{1}\right)}{x_{1}}+k_{2}\frac{\left(t_{3}-t_{2}\right)}{x_{2}}+k_{1}\frac{\left(t_{4}-t_{3}\right)}{x_{1}}$$

(2) $$q=h_{c}(t_{4}-t_{c})+\varepsilon \sigma (T_{1}-T_{c})$$

(3) $$q=h_{h}(t_{h}-t_{1})+\varepsilon \sigma (T_{h}-T_{4})$$

where q is the heat flux; $k_{1}$ and $k_{2}$ are the thermal conductivities of MR and CFF; $t_{1}$~$t_{4}$ are the temperatures of the surface and interlayers of the composite sandwich structure; $T_{1}$ and $T_{4}$ are the absolute temperatures of the hot and cold surfaces of the composite sandwich structure; $h_{h}$ and $h_{c}$ are the convective heat transfer coefficients of the hot and cold side surfaces, respectively; and ε is the blackbody emissivity of MR, which is the Stefan–Boltzmann constant, 5.67 × 10^−8^ W·m^−2^·K^−4^. The specimens are meshed with a mesh type of DC3D8. This allows the variation in the cold-surface temperature to be obtained.

## 4. Results and Discussion

### 4.1. Thermal Stability and Flame Retardancy

**Figure 3:**
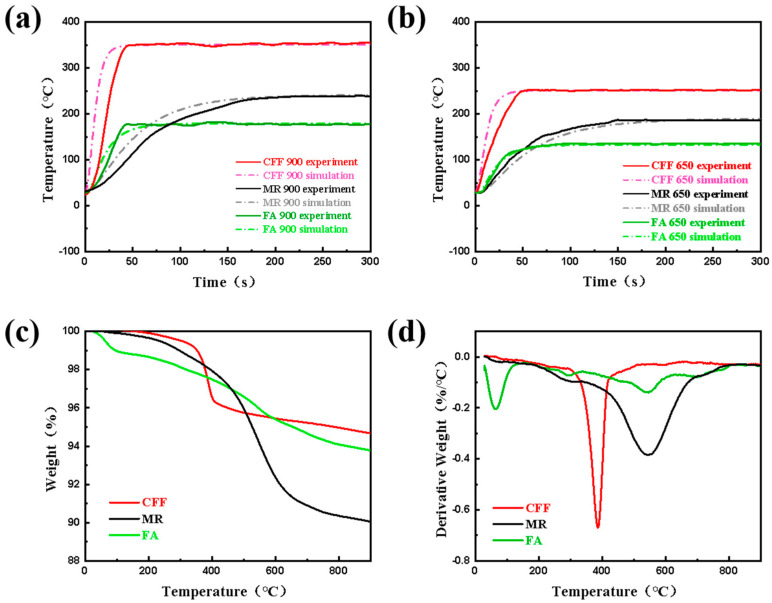
Experiments and simulations of the cold side temperature of MR, CFF, and FA at 900 °C (**a**) and 650 °C (**b**) and TGA (**c**) and DTG (**d**) images at 900 °C.

The temperature analysis in this section focuses on the cold side temperature variation in the constructed material. Figure 3a shows the cold-surface temperatures and the simulation results of MR, FA, and CFF with a thickness of 3 mm at 900 °C. Table 3 shows the comparison between their experimental and simulated values. Through Table 3, it can be found that although there is a deviation between the simulated values and the experimental results, it does not affect the overall trend of a rise in the cold side temperature of the material.

It can be seen that during the initial stage of the experiment, the CFF has a faster temperature rise rate and reaches a steady-state temperature in about 50 s. Due to the significant weight loss of the materials at approximately 400 °C (Figure 3c,d), which indicates the thermal decomposition of the organic binders within them, the structural integrity of the CFF is compromised, leading to an increase in the heat flow path. Under single-side flame heating, the adhesive on the surface of the material is decomposed significantly, while the pyrolysis degree of the organic matter in the deeper part is small and some matrix remains [33]. The decomposition of the surface organic substances provides channels for heat flow penetration, resulting in a faster fluid flow velocity and thus a faster temperature rise rate for the CFF. Since damage caused to materials was not considered during simulation process and thermal decomposition involves different reaction stages and mechanisms, there exists a slight deviation between the initial simulation results and the experimental results. When the cold-surface temperature reaches around 350 °C and tends to stabilize, better fitting occurs, as the heat transfer inside the materials mainly occurs through convection and radiation.

Similarly, during the initial heating stage, the temperature of the FA rapidly increased and reached its maximum value after 50 s. There were discrepancies between the simulated and experimental results during this stage. Heat conduction was mainly responsible for the heat transfer in the early stages of heating, but as temperature increased and forced convective heat began to exist, convective heat transfer and thermal radiation became the main modes of heat transfer [32]. According to the TGA (Figure 3c,d), FA exhibited a 6% mass loss over the entire temperature range, attributed to the high-temperature decomposition of its internal binders. The simulation results deviated from the actual results because of these factors. When thermal equilibrium was reached at a temperature of 185 °C, with stable pyrolysis reactions inside the material, the steady-state simulation results matched the experimental data well. Although aerogels have excellent insulation properties, they are brittle and require complex preparation processes with higher costs [34,35,36,37]. Therefore, they are not preferred insulation materials in this study.

The temperature rise rate of the MR is the slowest among the three materials, mainly because MR has a layered structure and there is no complete contact between the layers of the mica material. The gaps between layers create thermal resistance, resulting in a much lower solid thermal conductivity compared to its thermal radiation, thus leading to a slower temperature rise rate [13,20,24,25,38]. The MR used in this study primarily consists of mica paper and glass fiber laminated together using organic silicone resin as the adhesive. As a result, significant mass loss is observed within the temperature range of 900 °C, reaching up to 10% (Figure 3c,d). The most substantial mass loss occurs between 500 and 700 °C, primarily due to the decomposition of the organic silicone resin. However, since the resin content constitutes only 10% of the MR, this mass loss has a minimal impact on the structural integrity of the mica. The deviation in early-stage fitting can also be attributed to the decomposition of the organic substances within the material. According to multi-layer reflection principles, layered structures have good near-infrared reflectivity [39,40]. When thermal radiation reaches the surface of an object, it undergoes reflection, transmission, and absorption processes [19,21,24,25,39]. The various mechanisms involved in thermal radiation are also one reason for the simulation errors seen. Multi-layer structures effectively hinder the transfer of thermal radiation; therefore, it takes about 200 s for the MR to reach a steady state at a temperature of 250 °C, with its insulation performance falling between that of the CFF and FA materials.

Compared to the experimental data at 900 °C, it is not difficult to observe that the temperature rise trend seen in the material heated to 650 °C is more gradual. This is mainly due to the lower temperature resulting in a decrease in the heat flux density and a reduced impact of thermal shock on the material, leading to significantly improved fitting between the simulation results and experimental results.

**Figure 4:**
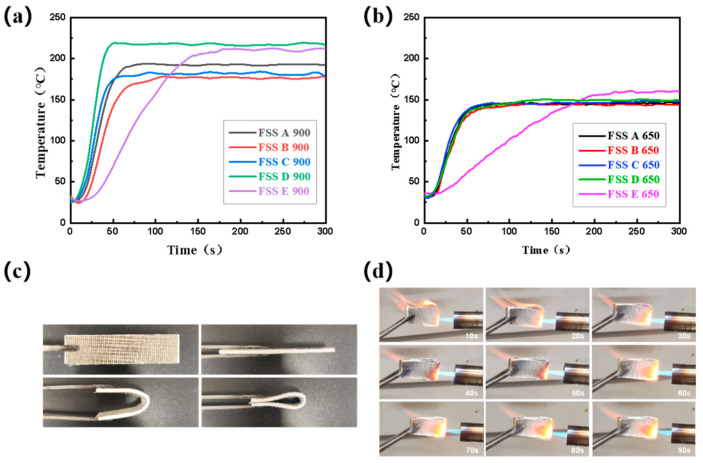
Cold-surface temperature of FSSs at 900 °C (**a**) and 650 °C (**b**); FSSs’ flexibility (**c**) and fire resistance (**d**).

The cold side temperature rise curves of FSSs at 900 °C are shown in Figure 4a. The cold side temperature of FSS A remains stable at around 190 °C, while testing on individual materials indicates that the steady-state temperature of 3 mm FA at 900 °C is approximately 185 °C, while for CFF it is about 350 °C. This suggests that the sandwich structure can significantly improve the insulation performance of CFF and achieve insulation effects comparable to FA, with a minimal difference in thickness.

When the thickness of the CFF in the sandwich structure is consistent, the influence of the MR the on overall insulation performance of the FSS can be observed through changes in the cold side temperatures of FSS B, FSS C, and FSS D. It can be seen that when the thicknesses are equal, a thicker MR leads to a better insulation performance for the sandwich structure. In terms of overall thickness, compared to FSS C, FSS B has an additional MR thickness of 0.3 mm and achieves a similar steady-state temperature ranging from 170 to 175 °C. The steady-state temperature of the structure FSS D is 220 °C. While its MR is only 0.06 mm thinner than that in FSS C, this produces a temperature difference of about 45 °C. The reason for this temperature difference is that the heat flow destroys the mica protective layer in FSS D and makes direct contact with the CFF located in the core layer, resulting in a brittle fracture of the ceramic fiber in the CFF, expanding the heat flow transmission path and reducing the obstruction of heat radiation so that the overall cold-surface temperature of FSS D is increased [35,36,41].

The experimental results of FSS C and FSS E show that when the total thickness of the sandwich structure is the same, a higher proportion of CFF leads to a better overall thermal insulation performance, indicating that CFF is the main component responsible for insulation. A higher proportion of MR leads to a smoother temperature rise trend in the composite sandwich structure but decreases its overall thermal insulation performance, suggesting that MR mainly shields against the heat flow’s impact and protects the integrity of the CFF structure.

Due to the destruction of the mica protective layer in FSS D at 900 °C, this work further explored the thermal insulation properties of FSSs at 650 °C (Figure 4b). It can be seen that similar to the temperature rise trend in the single materials, the overall temperature rise trend of the sandwich structure is less extreme at 650 °C. The steady-state temperature of FSS E is around 170 °C, while for all other structures except FSS E it ranges from 130 to 150 °C, with small temperature differences. This indicates that FSS D can be used as a protective structure at 650 °C. In addition to their excellent thermal insulation performance, FSSs also possess good flexibility and outstanding non-flammability. Even after folding, their structural integrity remains intact (Figure 4c). Moreover, the mica protective layer in the FSS has some heat shielding effect as it does not ignite even after being heated with fire for 90 s (Figure 4d).

### 4.2. Analysis of the Heat Insulation Mechanisms

#### 4.2.1. Structure Variations in Ceramic Fiber Felts

**Figure 5:**
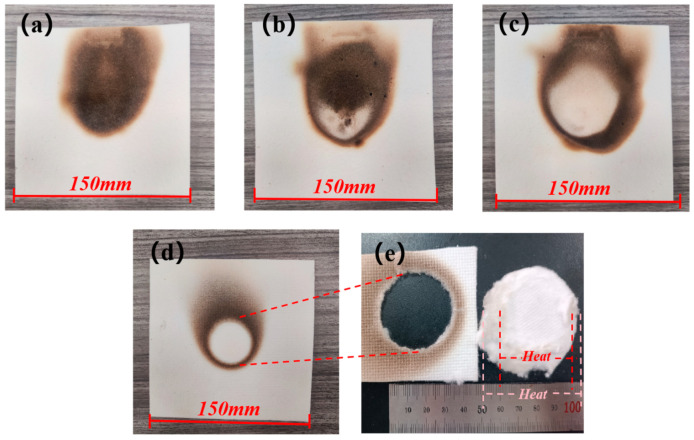
Macroscopic images of the CFF in FSS B (**a**), FSS C (**b**), FSS D (**c**), and CFF (**d**,**e**) directly heated at 900 °C.

Macroscopic images of the CFF in FSS B, FSS C, and FSS D, as well as the CFF alone, under the 900 °C one-sided heating experiment are shown in Figure 5. Figure 5a shows that the central region of the CFF in FSS B is completely black due to some oxidation–reduction reactions occurring within the organic material of the CFF; thus, the excellent thermal protection performance of the 0.3 mm mica layer is confirmed. In FSS C, a small white area can be observed in Figure 5b, which indicates the complete decomposition of the organic material due to the high temperature, but this does not significantly affect the overall insulation performance of the sandwich structure. However, Figure 5c displays that under the protection of only a 0.12 mm MR in FSS D, a large area of the central region of the CFF turns completely white, indicating that the protective effect of the 0.12 mm MR is minimal. Moreover, it can be seen that heat diffusion areas for the CFF in FSS B, FSS C, and FSS D are significantly larger than those when it is directly heated without any protective structure. This suggests that a heat flow perpendicular to the mica changes its direction inside mica during transmission [41,42]. After being exposed to a burning temperature of 900 °C without any protective layer, the unprotected CFF turns completely white on contact with the heat source (Figure 5d) and there is direct detachment in the middle of the white area (Figure 5e). The detachment range is smaller on one side close to heat source, while it is larger on another side; this indicates that heat only diffuses within a small range inside the CFF and detachment occurs because the excessive temperature causes a complete decomposition of the adhesive used for connecting the ceramic fibers inside the CFF, with the brittle fracture of the ceramic fibers supporting the overall structure caused by the presence of an impact force.

#### 4.2.2. Microscopic Morphology of Ceramic Fiber Felts

**Figure 6:**
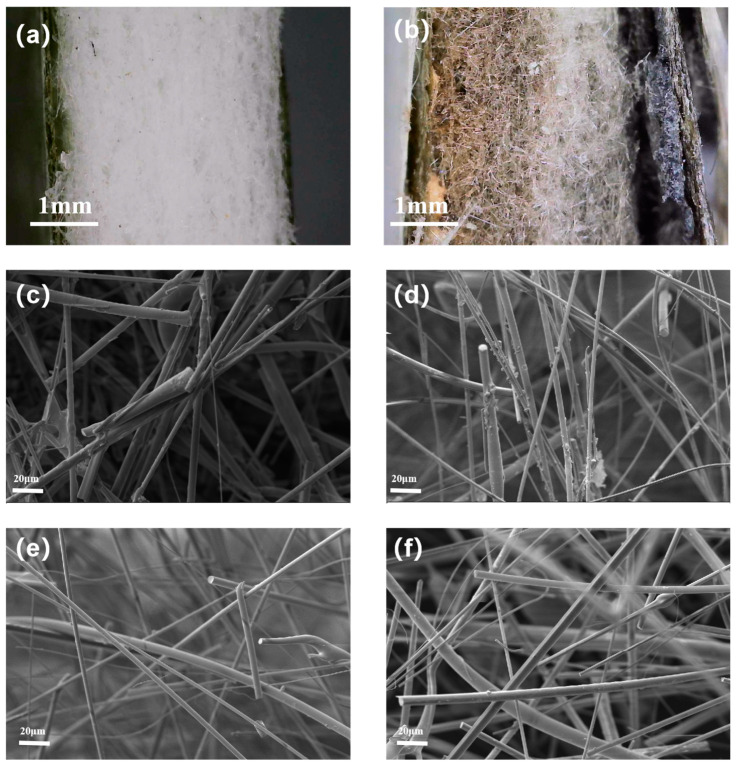
Microscopic images of FSS D before (**a**) and after (**b**) firing; SEM images of CFF in FSS B (**c**), FSS C (**d**), FSS D (**e**) heated at 900 °C and CFF (**f**) directly heated at 900 °C.

The thermal insulation performance of the CFF is closely related to its own microstructure, so we characterized the microstructure of the CFF under different protective states. Figure 6 shows the microscopic images of the sandwich structure and the SEM morphology of the CFF in the composite sandwich structures FSS B, FSS C, and FSS D, as well as in directly fired CFF. It is evident that compared to the FSS D before firing (Figure 6a), the mica layer after firing (Figure 6b) shows significant carbonization while still retaining its complete structure. This demonstrates the strong thermal shielding capability of the mica layer. On the other hand, the ceramic fiber felt displays color stratification and its right side is white, indicating that the organic matter in the material is almost completely decomposed and part of the fiber matrix is destroyed. With the increase in thickness, the degree of damage to the fiber felt decreases, indicating that even if the side close to the heat source is destroyed, it still has a certain ability to provide thermal protection.

There are a large number of surface treatment agents remaining on the fiber surfaces in FSS B and FSS C shown in Figure 6c,d, and with the decrease in the protective layer thickness, it is obvious that the content of the surface treatment agents on the fibers gradually decreases, leading to an increase in damage to the internal structure of the CFF. The structure inside the CFF in FSS D, shown in Figure 6e, has almost no attachments on the fibers, which is similar to the microscopic morphology of the unprotected structure shown in Figure 6f. The surface treatment agent was burned off, causing many through-holes inside the material, significantly increasing thermal convection within the three-dimensional pores [6,30,43,44]. This confirms that the poor thermal insulation effect in structure FSS D is due to the partial heat flow damaging the MR and thus compromising the integrity of the CFF structure.

#### 4.2.3. FT-IR of CFF and MR

**Figure 7:**
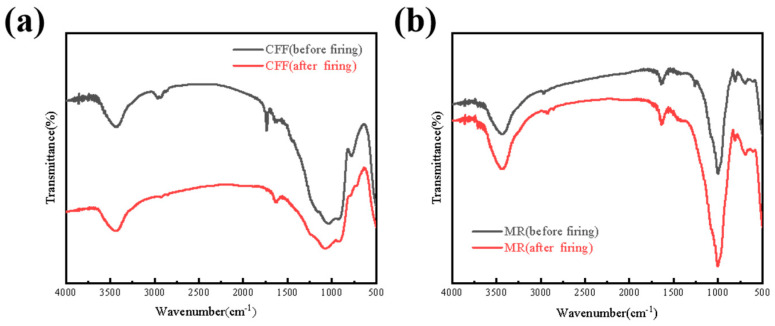
FT-IR spectra of CFF (**a**) and MR (**b**).

Figure 7 shows the FT-IR spectra of the CFF and MR. The infrared spectrum of the CFF shows that the -OH absorption peak appears at 3400 cm^−1^ and the Si-O absorption peak appears at 1100 cm^−1^, and there is no significant change before and after firing, indicating that the CFF has a certain amount of thermal stability. However, the C-H bond at 2900 cm^−1^ and the C=O bond at 1800 cm^−1^ disappear in the infrared spectrum after firing, indicating that organic compounds such as the binder inside the CFF are burned off, the structural integrity of the CFF is damaged, and the heat flow path is increased, thus reducing its heat insulation. It can be seen from the infrared spectrum of the MR that the strong absorption peak near 1000 cm^−1^ usually corresponds to the stretching vibration of the Si-O bond, while the absorption peak near 3400 cm^−1^ is related to the stretching vibration of the -OH bond. There is no significant change in the infrared spectrum before and after burning [44], which proves the unique thermal protection ability of mica materials.

### 4.3. Heat Transfer Mechanism of Sandwich Structures

**Figure 8:**
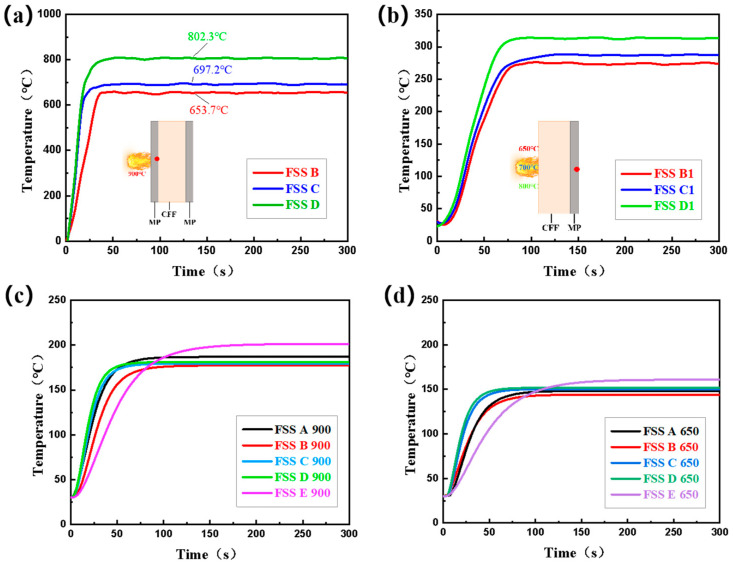
The temperature of the sandwich layer (**a**) of the composite sandwich structures at 900 °C and the temperature change in the cold surface of the FSSs (**b**) when one side is unprotected. Numerical simulation of the rise in the cold-surface temperature of the composite sandwich structures at 900 °C (**c**) and 650 °C (**d**).

Figure 8 shows the temperature of the composite sandwich structure and the numerical simulation of the rise in the cold-surface temperature of the composite sandwich structure at 900 °C and 650 °C. In order to verify the main role of the mica protective material in the composite sandwich structure, the structures FSS B, FSS C, and FSS D, which have the same core thickness, were tested and analyzed. First, the temperature of the three sandwich structures on the side near the heat source at 900 °C was tested; the red dot in Figure 8a is the temperature measurement point. The results show that the steady temperature of a flame kept at 900 °C when it passes through MRs of 0.3 mm, 0.15 mm, and 0.12 mm is 653.7 °C, 697.2 °C, and 802.3 °C, respectively.

By removing the protective structure on the side of FSS B, FSS C, and FSS D, as shown in the model diagram in Figure 8b, and directly heating the remaining structure with the corresponding three temperatures, respectively, it can be seen that the final steady-state temperature is much higher than the result in Figure 3a. The main function of the mica layer is thermal shielding, which can reduce thermal shock while shielding most of the heat from and protecting the integrity of the core layer structure. Compared with the CFF damaged under thermal shock, the complete CFF extends the heat transfer path and can give full play to the advantages of its porous structure. The pores inside the material are combined with the mica protective material to form a hollow structure [6,45,46], which greatly blocks heat transfer and improves the overall thermal insulation performance of the composite sandwich structure.

Figure 8c,d show the simulation results of the rise in the cold-surface temperature of the composite sandwich structures at 900 °C and 650 °C. It is not difficult to find that the simulation results of the composite sandwich structures at 650 °C are not much different from the experimental results. Although the steady-state temperature is not completely consistent, the deviation is small. The reason for the deviation in the simulation of FSS D is that the 0.12 mm MR cannot withstand the impact of the 900 °C heat source, and part of the heat flow penetrates the MR directly to reach the CFF, which further verifies that the 0.12 mm MR in the FSS D structure has a poor protective effect on the CFF at 900 °C.

**Figure 9:**
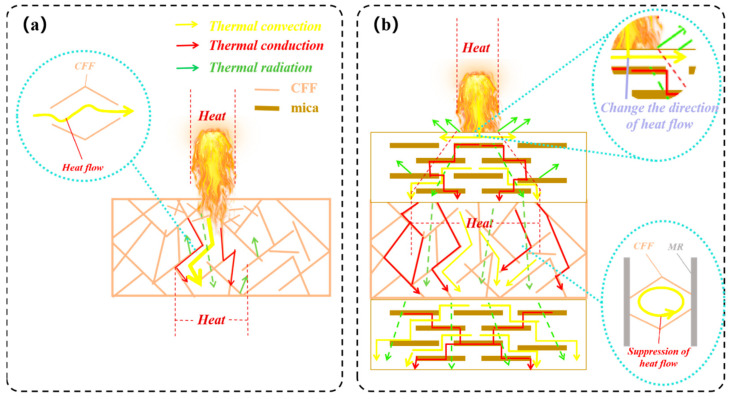
Schematic diagram of heat transfer in CFF (**a**) and heat transfer within FSSs (**b**).

Figure 9 is a schematic diagram of the heat transfer inside the CFF alone and an FSS material. The flexible mica protective layer not only shields against flames but also effectively blocks heat transfer due to its poor heat conduction properties perpendicular to the layer’s direction. At high temperatures, microcracks and holes inside mica materials expand, creating more new cracks, and the air in these cracks and holes increases the paths for heat transfer, thereby reducing the thermal conductivity of the material. Consequently, this provides protection for the porous structure of the CFF. When the heat flows into the ceramic felt, the solid skeleton and pore structure of the ceramic felt can absorb, reflect, and scatter the radiated heat energy through, thereby reducing the transfer of heat radiation. In this sandwich structure, there are a variety of heat transfer mechanisms at work, including gas-phase heat transfer, solid-phase heat transfer, and radiation heat transfer; these different heat transfer methods may affect each other, forming a coupling heat transfer effect so as to achieve collaborative heat insulation.

## 5. Conclusions

The MR//CFF//MR sandwich structure, under unilateral flame heating conditions, demonstrates an exceptional thermal insulation performance through the synergistic coupling of multiple heat transfer mechanisms within its layered and porous materials. At a hot-surface temperature of 900 °C, the cold-surface temperature of the sandwich structure with a 0.3 mm MR and 3 mm CFF was only 175 °C, which is significantly lower than the 350 °C observed for a standalone 3 mm CFF under the same conditions. This remarkable reduction in temperature highlights the effectiveness of the MR layer in shielding against flames and blocking heat transfer, while the porous structure of the CFF enhances insulation by increasing the number of heat transfer paths and scattering radiated heat. The unique combination of these materials not only improves thermal insulation but also ensures structural integrity under high-temperature conditions. By effectively mitigating thermal runaway propagation, the proposed sandwich structure can contribute to the development of safer battery modules, particularly in applications such as electric vehicles and portable electronics, where thermal management is critical. This study advances the field of thermal insulation materials and provides a foundation for future innovations in battery safety and energy storage technologies.

## Figures and Tables

**Table 1:** Density and thermal properties of MR, FA, and CFF.

Material	Density (g·cm^−3^)	Thermal Conductivity (W·m^−1^·k^−1^)	Heat Capacity (J·kg^−1^·K^−1^)
MR	1.80	0.220	836
FA	0.20	0.023	970
CFF	0.19	0.036	1000

**Table 2:** The thickness of each layer of the FSS and the total thickness of the sandwich structure.

Samples	MR (mm)	CFF (mm)	MR (mm)	Total Thickness (mm)
FSS A	0.3	2.5	0.3	3.1
FSS B	0.3	3	0.3	3.6
FSS C	0.15	3	0.15	3.3
FSS D	0.12	3	0.12	3.24
FSS E	0.9	1.5	0.9	3.3

**Table 3:** Experimental and simulated data of the time and steady-state temperature of the materials at 900 °C.

	CFF		MR		FA	
	t (s)	T (°C)	t (s)	T (°C)	t (s)	T (°C)
EXP	50	357	177	223	47	183
SIM	31	350	150	220	73	185

## Data Availability

The original contributions presented in the study are included in the article, further inquiries can be directed to the corresponding author.
